# Caste-specific nutritional differences define carbon and nitrogen fluxes within symbiotic food webs in African termite mounds

**DOI:** 10.1038/s41598-019-53153-x

**Published:** 2019-11-13

**Authors:** Risto Vesala, Laura Arppe, Jouko Rikkinen

**Affiliations:** 1 Finnish Museum of Natural History, University of Helsinki, Helsinki, Finland; 2 Organismal and Evolutionary Biology Research Programme, Faculty of Biological and Environmental Sciences, University of Helsinki, Helsinki, Finland

**Keywords:** Stable isotope analysis, Tropical ecology, Ecological networks

## Abstract

Fungus-growing termites of the genus *Macrotermes* cultivate symbiotic fungi (*Termitomyces*) in their underground nest chambers to degrade plant matter collected from the environment. Although the general mechanism of food processing is relatively well-known, it has remained unclear whether the termites get their nutrition primarily from the fungal mycelium or from plant tissues partly decomposed by the fungus. To elucidate the flows of carbon and nitrogen in the complicated food-chains within the nests of fungus-growing termites, we determined the stable isotope signatures of different materials sampled from four *Macrotermes* colonies in southern Kenya. Stable isotopes of carbon revealed that the termite queen and the young larvae are largely sustained by the fungal mycelium. Conversely, all adult workers and soldiers seem to feed predominantly on plant and/or fungus comb material, demonstrating that the fungal symbiont plays a different nutritional role for different termite castes. Nitrogen stable isotopes indicated additional differences between castes and revealed intriguing patterns in colony nitrogen cycling. Nitrogen is effectively recycled within the colonies, but also a presently unspecified nitrogen source, most likely symbiotic nitrogen-fixing bacteria, seems to contribute to nitrogen supply. Our results indicate that the gut microbiota of the termite queen might be largely responsible for the proposed nitrogen fixation.

## Introduction

Insects consuming vegetative plant parts, such as stems, roots or leaves, face two fundamental problems. Firstly, plant cell walls consist of a complex mixture of cellulose, hemicelluloses and lignin that makes them highly resistant to degradation^1^. Digestion of such compounds requires a set of different enzymes and, in many cases, symbiotic prokaryotes, bacteria or fungi are needed for their production^1–3^. Secondly, the nutritional value of many supportive plant tissues, especially wood, is extremely low^4^. Due to their high carbon to nitrogen (C/N) ratio, large amounts of plant biomass must be processed to gain sufficient nitrogen especially for reproduction and to adequately support growth of developing instars^4–7^.

Fungus-growing termites (Macrotermitinae, Termitidae, Blattodea) of the Old World tropics utilize symbiotic fungi of the genus *Termitomyces* (Lyophyllaceae, Basidiomycota) to overcome these challenges. Termites collect plant litter from the nest surroundings and provide it for the fungal symbiont that effectively decomposes lignocellulose in specific compost structures (fungus combs) that serve as a type of ‘external rumen’ for the insect hosts^8^. Symbiosis between Macrotermitinae and *Termitomyces* is believed to have evolved in African rain forests ca. 30 million years ago, but it has since spread to the dry savannas where the fungus-growing termites and their *Termitomyces* symbionts have become ecological keystone organisms^9–12^. Especially mound building termites of the genera *Macrotermes* and *Odontotermes* can control their nest internal climates that allow effective plant decomposition to take place year-round even in the most arid savanna ecosystems^12–15^.

The main benefits of the symbiotic relationship between fungus-growing termites and their symbionts stem from the ability of the fungal mycelium to produce enzymes needed for effective plant decomposition^8,16–24^ and to enrich nitrogen from the nutrient poor plant biomass^5,20,25,26^. The role of *Termitomyces* has been suggested to be differently balanced between these two functions within different termite genera: in the genus *Macrotermes* the primary role of the fungal symbiont has been suggested to be lignin degradation, thus making plant material more digestible for the insects, whereas in several other genera of Macrotermitinae the highly nutritious fungal mycelium itself is believed to represent a primary food source for the termites^26^. As the collective food processing of fungus-growing termites, however, is highly complex, and includes several different termite castes each with different roles and functions^27^, more detailed studies on nest internal nutrient fluxes are needed to comprehensively understand the significance of the symbiotic fungus for colony nutrition.

**Figure 1:**
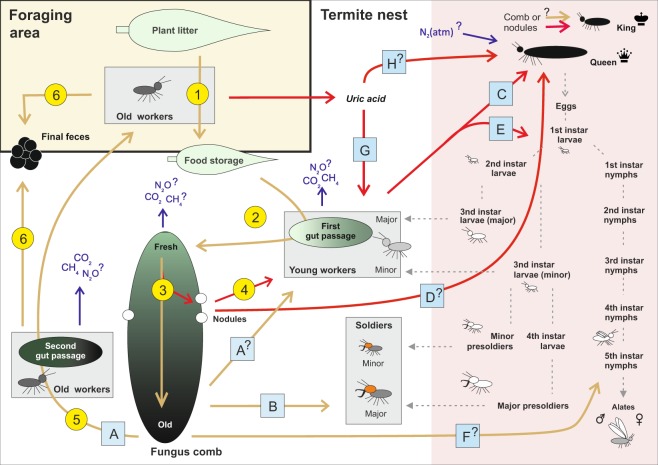
Food processing and utilization within a *Macrotermes* colony. Yellow lines represent biomass with high (>10) and red lines with low C/N ratio (<10). Dotted lines illustrate development of sterile instars and sexual alates^85^. Circled numbers outline the symbiotic food processing based on previous literature: (1) Termite foragers that typically are old major workers collect plant litter from the environment and transport it into nest food storages^29,31,32^. (2) Young workers eat the collected material and defecate it into upper sections of fungus combs (first gut passage)^29,31,33^. (3) Partly decayed plant biomass becomes substrate for the *Termitomyces* mycelium growing within the fungus combs^31,86^. Degradation of plant matter in combs typically proceeds from top to bottom^87^. (4) *Termitomyces* produces spherical nodules that contain asexual fungal spores (conidia)^53^. Nodules are consumed by young workers leading to the inocluation of new plant material with *Termitomyces* conidia during the first gut passage^31,53^. In addition, the fungal enzymes act synergistically with termite endogenous and gut bacteria derived enzymes during the first gut passage and within the fresh parts of the fungus combs^8,16,18,19,23,35^. (5) Finally, the oldest parts of the fungus combs containing plant residue and senescent *Termitomyces* mycelium are eaten by old workers (second gut passage)^29,31^. (6) By the end of the second gut passage most nutrients within the plant material have been utilized and the refuse matter is eventually deposited as final feces into dump sites^29,86^. Letters in squares, demonstrating the utilization of different food sources by each caste, are discussed in the text.

Much of the currently available information on mechanisms of symbiotic food processing among fungus-growing termites have been gained from incipient laboratory colonies of three African *Macrotermes* species (*M*. *michaelseni*, *M*. *subhyalinus* and *M*. *bellicosus*) and two Oriental *Odontotermes* species (*O*. *formosanus* and *O*. *obesus*). In all these species food processing is carried out in a similar manner by the termite workers with an elaborate age-dependent division of labor^28–34^. Biochemical and genomic studies have provided additional information of the enzymes involved in the degradation of plant matter^16,17,19,21,23,24,35,36^. A schematic roadmap of the complicated food processing chain, which for example involves two consecutive gut passages through different-aged workers, is illustrated in Fig. 1.

Although only mature termite workers are involved in the food processing, *Macrotermes* colonies always include also several other castes: a queen and king, major and minor soldiers, asexual larvae representing different instars, and seasonally produced sexual nymphs that develop into swarming alates^27^. All these castes are thought to be completely dependent on care of workers because they do not have access to food sources and/or are not able to eat without assistance^27^. The reproductives and developing castes are thought to be fed with liquid excreted from the labial glands of workers (i.e. stomodeal trophallaxis), whereas the soldiers are supplied with pieces of fungus comb material^29,31^. As a sufficient supply of nitrogen is crucial especially for the queen which produces 5000–18,000 eggs per day^37^, the food provided for her must be highly nutritious. The nitrogen content of fungal mycelium and especially the nodules produced by the fungus is high compared to the foraged plant litter and fungus comb material^5,25,38^. Hence, it seems reasonable to presume that the food provided for the reproductives and developing instars would be at least partially of fungal origin. However, no direct conformation of the nutritional role of *Termitomyces* has as yet been made for any *Macrotermes* species.

Stable isotope analysis of carbon and nitrogen are widely used to define animal diets and to elucidate elementary fluxes in food webs^39–41^. This approach has also been applied in research of fungus-growing termites^22,26,42–46^, but no studies on the actual transfer of carbon and nitrogen within the social and symbiotic networks of termite colonies have been published so far. In this study we explored stable isotope signatures of carbon and nitrogen to elucidate the nutritional role of the *Termitomyces* fungus for different termite castes and age-groups of two Kenyan *Macrotermes* species (*M*. *michaelseni* and *M*. *subhyalinus*). In order to understand how fungal degradation affects the isotopic composition of foraged plant matter and to follow the subsequent food processing within termite mounds step by step, we sampled all nodes of the symbiotic food chain, starting from dead plant matter in the nest environment and in termite food storages, fresh and old parts of fungus combs, fungal nodules, all different termite castes, and finally the feces deposited into peripheral tunnels of the nest.

## Results

### C/N balance and the enrichment of nitrogen during food processing

**Figure 2:**
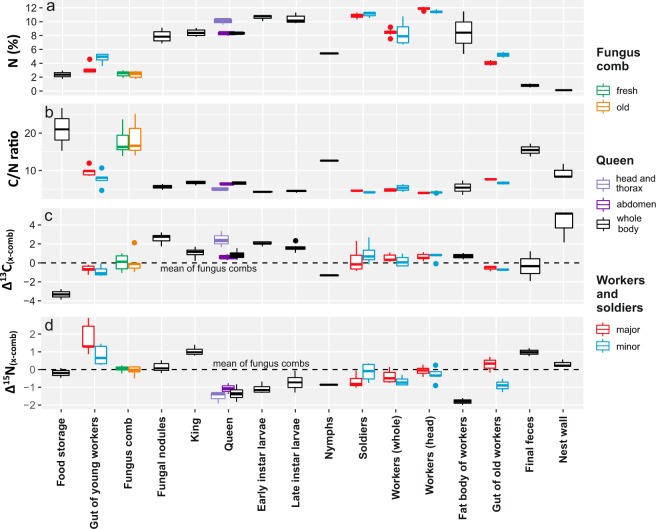
Nitrogen enrichment and differences in C/N ratios and stable isotope values of different components of the plant-fungus-termite food chain (data pooled from all studied termite colonies). (**a**,**b**) Nitrogen content and C/N ratios of analyzed specimens representing different termite castes and nest components. (**c**,**d**) Difference of δ^13^C and δ^15^N values (denoted as Δ^13^C and Δ^15^N) of analyzed termites, their body parts and nest components when compared with average values obtained from fungus combs of each colony. Whiskers show 1.5 IQR. Results from the distinctive minor workers with whitish abdomens from colony TR400 were omitted from the figure because of their exceptionally high nitrogen content (18.5%) and low ^15^N values (Δ^15^N_x-comb_ = −4.0).

Nitrogen content of analyzed plant matter ranged from a minimum of 0.4% (wood of *Acacia mellifera*) to a maximum of 4.8% (*Acacia tortilis* leaves) resulting in C/N ratios of 112.5 and 10.4, respectively (Supplementary Table S1). Nitrogen contents were on average highest in leaves of woody plants and lowest in wood (Table 1). C/N ratios of grasses were typically lower than in wood but higher than in the leaves of trees and shrubs (Table 1). Nitrogen contents and C/N ratios of plant matter in termite food storages were comparable to levels measured in surrounding vegetation (Fig. 2a,b).

**Table 1:** Stable isotope values (δ^13^C and δ^15^N), carbon and nitrogen contents and C/N ratios (mean ± SD) of plant material collected from the study area. For more detailed information on the plant specimens, see Supplementary Table S1.

	Specimens	δ^13^C	δ^15^N	C cont. (%)	N cont. (%)	C/N ratio
Woody plants						
*leaves*	11	−27.7 ± 1.42	5.9 ± 2.44	42.7 ± 5.42	2.8 ± 0.94	16.8 ± 4.81
*wood*	11	−26.9 ± 1.73	2.6 ± 2.51	45.7 ± 1.75	1.0 ± 0.36	52.5 ± 24.44
*bark*	6	−26.9 ± 1.47	2.4 ± 4.21	42.4 ± 8.51	1.3 ± 0.82	50.1 ± 34.97
Grasses						
*dry leaves*	16	−13.4 ± 0.84	4.9 ± 1.77	40.7 ± 1.47	1.3 ± 0.66	38.7 ± 16.8
*fresh leaves*	5	−14.1 ± 0.47	6.1 ± 2.07	40.7 ± 1.28	1.7 ± 0.93	30.8 ± 16.82

Notable enrichments in nitrogen content were not detected when moving from food storages to fungus combs or between fresh and senescent parts of fungal combs (Fig. 2a). Conversely, a clear enrichment (>5%) of nitrogen was observed between fungus combs and fungal nodules, with the mean nitrogen content being 7.9% (SD = 0.78) in fungal nodules (Fig. 2a). Excluding the fatty body parts of the reproductive individuals, all tissues of all termite castes had much higher nitrogen contents and lower C/N ratios than fungal nodules (Fig. 2a,b). The nitrogen content of eggs (analyzed from colony TR400) was exactly the same as that of the queen (8.5%). The highest nitrogen contents were generally measured from heads of major and minor workers, with the guts of the same individuals always having much lower contents of nitrogen (Fig. 2a). The distinctive group of minor workers with white abdomens (found in colony TR400) had exceptionally high nitrogen contents: 18.5% and 27.3% in entire bodies and fat body tissues, respectively. The lowest nitrogen contents within the termite nests were measured from final feces and chamber wall structures (Fig. 2a).

### Carbon stable isotopes

**Figure 3:**
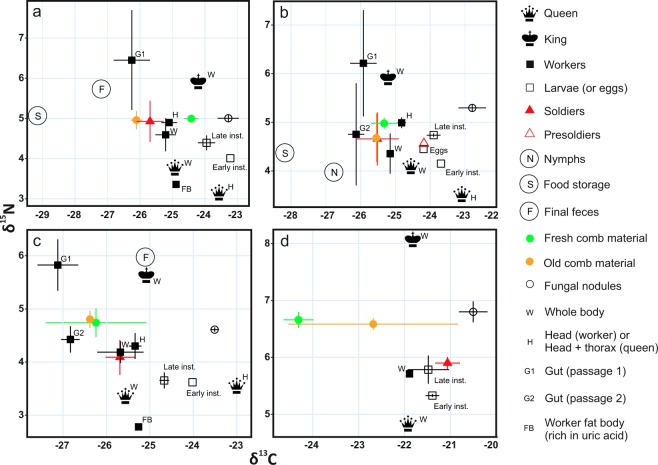
Carbon and nitrogen stable isotope values (mean with SD) of analyzed nest components in four *Macrotermes* colonies ((**a**) TR9, (**b**) TR400, (**c**) TR183 and d: MR1). Data for the distinctive minor workers with whitish abdomens found from colony TR400 (panel b) was omitted due to exceptionally low δ^15^N values (0.8‰), interfering with panel y-scale comparability.

Carbon stable isotope values (δ^13^C) of the grass specimens collected from the study area ranged from −14.7 to −11.9‰ and those of trees and shrubs from −29.3 to −22.3‰ (Table 1, Supplementary Table S1). The δ^13^C values of plant matter in termite food storages (<−28‰ in both cases) were equal to the lowest values obtained from woody plants of the same habitat (Fig. 3a,b). Notable increases in δ^13^C values took place both between food storages and fungus combs and between the combs and fungal nodules. Total enrichment of ^13^C from the plant matter of food storages to fungal nodules was 5–6‰ (Fig. 2c). Fungal nodules generally had the highest δ^13^C values of analyzed nest components (Fig. 3). Fresh and old sections of fungus comb did not differ consistently from each other in respect of their δ^13^C values. In most cases fresh comb material was more enriched in ^13^C than older material but within-nest variation was quite high (Figs 2c and 3).

Major differences were detected in δ^13^C values of different termite castes. Larvae were always more enriched in ^13^C than adult workers or soldiers (Figs 2 and 3). When analyzed separately, the head and thorax tissues of queens had higher δ^13^C values than entire bodies (Δ_head/thorax – whole body_ = 1.5–3‰, Figs 2c and 3). However, the abdomens of queens with lipids removed showed comparable δ^13^C values as head and thorax tissues (Supplementary Fig. S1). Early instar larvae were consistently more enriched in ^13^C than the later instar larvae (Figs 2c and 3). Soldiers generally exhibited similar δ^13^C levels to those measured from fungus combs, except in colony MR1 where both major and minor soldiers were unusually enriched in ^13^C (Fig. 3). Different tissues of workers had contrasting δ^13^C values. In workers the heads were much more enriched in ^13^C than the guts, which generally had more negative δ^13^C values than those recorded from the fungus combs. Final fecal material analyzed from colonies TR183 and TR9 showed either slight enrichment or depletion in ^13^C, respectively, when compared to the fungus comb values (Fig. 3).

### Nitrogen stable isotopes

Unlike in the case of carbon isotopes, there were no marked differences in the δ^15^N values of plant matter in food storages and fungal nodules (Fig. 2d). When compared to the mean δ^15^N values of fungus combs and nodules, all termite castes except the king were either at the same level or depleted in ^15^N (Fig. 2d). The lowest δ^15^N levels were found from queens and larvae (Figs 2d and 3). Even lower values were recorded from the white fat body tissue (presumably rich in uric acid^46,47^) that had accumulated in the abdomens of some termite workers (Fig. 2d). Minor workers with notably whitish swollen abdomens (found only from colony TR400) exhibited exceptionally low δ^15^N levels, i.e., 0.8‰ and −1.0‰ in entire bodies and fat body tissues, respectively.

The king consistently had a much higher δ^15^N value than any other termites sampled from the same nest (Fig. 3). Also final fecal material and the guts of young workers (corresponding with the first gut passage) were more enriched in ^15^N than other analyzed materials. In contrast, the guts of old workers (corresponding with the second gut passage) exhibited either comparable or lower δ^15^N values to those of fungus combs and nodules. The guts of major workers had consistently higher δ^15^N values than those of minor workers (Fig. 2d).

## Discussion

### Food selection and C/N ratios

In African savannas woody plants and grasses accumulate stable isotopes of carbon (^12^C and ^13^C) in different proportions, leading to characteristic, non-overlapping isotopic signatures that have been widely used to elucidate proportions of carbon derived from trees and shrubs or grasses in the diets of various savanna herbivores including termites^42,43,48^. Due to C3 photosynthesis the δ^13^C values of trees and shrubs typically range from −34‰ to −24‰, while those of C4 savanna grasses typically range from −15.9‰ to −11.0‰^49,50^. The low δ^13^C values found from the fungus combs of the three termite nests in Kasigau Road reveal that the insects had recently been foraging exclusively on trees and shrubs. Conversely, the colony in Mbula (MR1), with distinctly higher δ^13^C values, had probably also utilized grasses as a minor dietary component. Completely in line with this interpretation, grasses were very scarce at the Kasigau Road study site (woodland savanna with large sparsely distributed trees) while in Mbula (relatively dense bushland) grass litter was much more abundant during the sampling. Colonies in Mbula and Kasigau Road were also sampled during different seasons (either in the beginning or in the end of dry season, respectively) and, thus, the differences probably reflect also temporal variance in the availability of fresh litter.

A notable increase in nitrogen content and a corresponding decrease in C/N ratio from plant material to fungal nodules was evident in all studied termite colonies (Fig. 2a,b). Only a minor decrease in C/N ratios was observed from food storages to fungus combs (step 2/Fig. 1, and Fig. 2) but the *Termitomyces* mycelia were clearly able to effectively enrich nitrogen and allocate it into fungal nodules (step 3/Fig. 1, and Fig. 2). The nitrogen content of nodules (7–9%) is consistent with comparable studies^5,25,38,51,52^, and represents an approximately 4-fold enrichment of nitrogen from the source plant matter mean. Among the termites, the highest nitrogen content was found in the sterile castes (larvae, workers and soldiers). The lower nitrogen values of whole body samples of termite workers when compared to those of heads reflect the lower nitrogen content of their guts, mainly containing plant matter and fungal conidia (young workers) or fungus comb material and soil (old workers)^29,31,53^. Conversely, in the case of sexual castes, the lower nitrogen content is likely to mainly reflect their high abdominal fat contents^54,55^.

### Decomposition of plant biomass

We detected a remarkable increase in δ^13^C values from the stored plant biomass to the fungal nodules (Fig. 2c). The average enrichment of ^13^C from plant material to fungus combs was 3.0–3.5‰, and further 2.5–3.0‰ during the step from combs to nodules (steps 2 and 3 in Fig. 1). Comparable levels of ^13^C enrichment from food storages to the fungal nodules have been reported also in previous studies^46,56^. Discrimination against ^12^C is by no means restricted to *Termitomyces* but has been observed also in several other saprotrophic fungi^57–60^, and is thought to be due to favoring of heavy trioses (rich in ^13^C) during sugar uptake by the fungal cells^57^.

While the δ^13^C values of the fungus combs, nodules and all termites were well above those in plant material, the assumed sole source of carbon for the colony, isotopic mass balance necessitates that a sink of light carbon must exist in the nest ecosystem that was not captured by our sampling. Although the carbon dioxide emitted by *Macrotermes* colonies have been reported to have similar δ^13^C values to those of their fungus combs^44^, methane emissions of termite mounds can be highly ^13^C depleted, with observed δ^13^C values ranging from −45‰ to −66‰^44^. Methane production by archaea has been found to occur in the hindguts of most termite species including *M*. *subhyalinus*^61,62^. This provides a plausible mechanism of ^13^C enrichment during the transfer of plant matter to fungus combs (step 2 in Fig. 1). Recently, several species of saprotrophic fungi have been shown to produce methane in aerobic conditions, although the exact pathway remains unknown^63,64^. Presuming that also *Termitomyces* has this capacity, aerobic production of methane with low δ^13^C levels by the fungal mycelium within the fungus combs could balance the observed further ^13^C enrichment from the fungus combs to the nodules.

Contrary to carbon, the degradation of plant matter was not depicted in the isotopic composition of nitrogen, as plant matter, fungus combs and fungal nodules exhibited all very similar δ^15^N values (Fig. 2d). Comparable observations have also been made in studies of non-symbiotic saprotrophic fungi^59^. Against this background it is somewhat surprising that the guts of young termite workers which contained fresh plant material, exhibited much higher δ^15^N values than the plant matter in food storages, fungus combs or fungal nodules (Fig. 2d). The values were clearly higher than those recorded from the guts of the old workers and also higher than in any samples from the sterile castes. We suspect that the elevated δ^15^N levels of first passage guts is linked to the sedentary microbiota that resides in the digestive tracts of young workers^62,65^.

### Nutrition of termites

Although the high dietary potential of *Termitomyces* nodules is widely recognized, their actual contribution to termite nutrition remains unclear^5,8,25,33,66^. In a study comparing the worker castes of five different genera of Macrotermitinae, Hyodo *et al*.^26^ proposed that for the genus *Macrotermes* the nutritional role of *Termitomyces* would be indirect: the fungal symbiont degrades lignin and allows the termites to utilize cellulose and other compounds more effectively. Instead, in case of the other studied termite genera cultivated fungi were thought to rather serve as a direct food source for the colony^26^. However, inferences about the diet of mature workers should not be generalized to the level of whole colonies. Instead, the nutrition of all termite castes, including reproductive individuals and larvae with high levels of anabolism, should be addressed before drawing final conclusions about the role of the fungal symbiont in colony subsistence.

Based on our results, we propose that at the colony level the previously discussed roles of *Termitomyces* are not mutually exclusive, and that the symbiotic fungus in fact enhances termite nutrition both directly and indirectly. The δ^13^C systematics of the colonies studied clearly demonstrate that fungal tissue serves as food source for termites, but this is not the case for all castes within the colony. The distinctive ^13^C enrichment in fungal nodules compared to both plant matter and fungus combs allows us to track the dietary flow of fungus derived carbon in different termite castes, based on the fact that the δ^13^C values of animals closely resemble those of their diets^39^.

The similarity of δ^13^C values of adult major and minor workers to those of the fungus comb material (Figs 2c and 3), indicates that most of their nutrition was derived from plant material and/or fungus combs. This is in congruence with the suggestion that mature *Macrotermes* workers rely on fungus comb material as their main source of nutrition^22,26,33^ (Fig. 1A). However, it remains unclear if also young workers get their nutrition by eating senescent parts of fungus combs, or if their nutrition is a mixture consisting of plant material from food storages, with relatively lower, and fungal nodules, with relatively higher δ^13^C values compared to workers, as could be expected based on the prevailing hypothesis^53^.

The δ^13^C values of mature termite soldiers were also closely comparable with those of fungus combs. This was expected as also in previous literature *Macrotermes* soldiers have been reported to feed on senescent comb material^29,31,33^ (Fig. 1B). The soldiers of colony MR1 showed exceptionally high δ^13^C values (Fig. 3d). This anomaly was obviously linked to considerable within-colony variation in δ^13^C values of old fungus combs, which in turn, was likely caused by a recent change in the type of litter foraged by this colony. The isotopic compositions of insects tends to follow dietary changes with a lag of several days due to relatively slow turnover of chitin and other body tissues^67,68^.

The massive fatty abdomens of *Macrotermes* queens complicates comparisons of the whole body δ^13^C values of queens and adult sterile castes. Relative to dietary input and other biochemical fractions, lipids are known to display significantly lower δ^13^C values in animals, plants and micro-organisms^69^. Due to this lipid bias, the whole bodies and abdomens of the queens did not differ that much from workers and soldiers in terms of δ^13^C values, but the carbon isotope composition of head and thorax samples – more directly comparable to the biochemical makeup of the other mature castes – were always much higher than those of plant matter and fungus combs, corresponding closely with the isotope composition of fungal nodules (Figs 2c and 3). The workers of both *M*. *subhyalinus* and *M*. *michaelseni* have been reported to feed the queen with liquid excreted from labial glands^29,31^. Hinze *et al*.^33^ suggested that in *M*. *bellicosus* both labial gland secretions (Fig. 1C) and *Termitomyces* nodules (Fig. 1D) would be offered to the queen by two different age-groups of workers, but direct evidence of this is lacking. The high carbon isotope values of queen tissues obtained in our study unequivocally show that fungus combs cannot represent the main food source of the queens. Both the high head and thorax δ^13^C values of queens, and the universally recognized pattern of lipid ^13^C depletion relative to dietary input, necessitate that the principal food source has a δ^13^C value well above that of fungus combs, and thus indicates that the food of the queen is mainly derived from *Termitomyces*.

The same pattern of high δ^13^C levels was also seen in larvae representing sterile castes, with the early instars being consistently more enriched in ^13^C than the later instars (Figs 2c and 3). The observed gradual ^13^C depletion during larval development could indicate that the proportion of fungus derived compounds in diets of larvae decreases with increasing age (Fig. 1E). The sexual nymphs had markedly lower δ^13^C values than larvae of the sterile castes, and even lower than the fungus combs (Figs 2c and 3b). This reflects their high lipid content^54,70^ but also a food source that needs to be relatively depleted in ^13^C. Thus, we propose that the sexual instars which need to accumulate fat to supply energy for swarming and reproduction are mainly fed with fungus comb material (Fig. 1F).

### Nitrogen balance of termite colonies

Due to the very similar δ^15^N values of ambient vegetation (Table 1), stored plant litter, fungus combs and fungal nodules (Fig. 2d), stable isotopes of nitrogen cannot be used to further quantify the relative contributions of different dietary resources in the diets of different termite castes. However, several observations suggest complex and intriguing patterns of nitrogen systematics within termite colonies. The δ^15^N values of all termite castes except the king were lower than those of their assumed nitrogen sources (plant matter, fungus combs or nodules). Especially the queens exhibited very low δ^15^N values compared to any other biological material sampled from the nests (Figs 2d and 3). This pattern contradicts the usual scenario where animals tend to be more ^15^N enriched than their diets, giving rise to the phenomenon known as trophic enrichment, where the preferential retention of ^15^N in organism tissues is balanced out by ^15^N depleted phases leaving the body in form of sweat, urine and feces^40,71,72^.

Tayasu *et al*.^46^ observed similar ^15^N depleted pattern in *Macrotermes* workers and soldiers relative to their diets and proposed that it would be explained by the accumulation of uric acid (UA) in termite tissues. *Macrotermes* species and many other termites accumulate UA in fat body tissues of workers^6,46,47^. This highly nitrogenous compound is apparently recycled with the aid of uricolytic bacteria residing in termite hindguts through necrophagy or cannibalism^5,6,73^. As uric acid content has been found to correlate negatively with the δ^15^N values of *Macrotermes* workers^46^, this compound needs to be relatively depleted in ^15^N. We found white chalk-like material around the alimentary tracts of some dissected termite workers. As this material showed distinctively lower δ^15^N values than any other tissues that were analyzed (Figs 2d and 3), we suspect that it largely consisted of UA. Tayasu *et al*.^46^ reported that in *Macrotermes muelleri*, *M*. *gilvus* and *M*. *annandalei* uric acid was accumulated especially in old minor workers. Consistently with that, we found a few minor workers with notably whitish abdomens (Supplementary Fig. S3) that exhibited highly ^15^N depleted values compared to those of other workers. It is possible that these termites represented old individuals predestined for the upcoming dietary utilization of their tissues for the general benefit of the remaining colony.

Behavioral studies on *Macrotermes* colonies have shown that dead colony members are regularly eaten by the nest mate workers^31^. As the δ^15^N levels of animals generally reflect the δ^15^N values of their food^40^, regular consumption of relatively ^15^N depleted UA may act to decrease the δ^15^N values of those workers (Fig. 1G). However, the conspicuously low δ^15^N levels of all studied queens, could imply that the recycled UA is eventually utilized by this reproducing individual. Workers could supply the UA to the queen either directly in the form of fat body tissue (Fig. 1H) or by recycling the nitrogen through their salivary glands (Fig. 1G + C). Uric acid contains ca. 33% of nitrogen which potentially makes it a highly valuable food source for the continuously egg-laying queen. Urate cells have not been found from the fat bodies of physogastric *Macrotermes* queens^74^, and thus, the accumulation of UA per se is not likely to play a significant role in the ^15^N depletion of the queens.

However, while uric acid recycling may be a significant mechanism in accounting for the inversed pattern of “trophic depletion” in various termite castes, it does not resolve the overall isotopic imbalance between measured inputs, termite biomass and known outputs. As a whole, comparison between the ^15^N depleted termite biomass and the plant material harvested, clearly indicates that, either nitrogen with a relatively high δ^15^N value must be lost from the system, or an additional, still unrecognized flux of isotopically light nitrogen must enter the termite food web.

Our results demonstrated that only final feces and termite kings are clearly more enriched in ^15^N than the fungus combs and nodules (Fig. 2d). While a single king with a high δ^15^N value obviously cannot maintain the isotopic mass balance of innumerable other colony members with low δ^15^N values, a relatively high proportion of heavy nitrogen could be deposited in fecal dumps. However, as the nitrogen content of fecal material was on average 10 times lower than in termite biomass (Fig. 2a), massive amounts of fecal material would be needed to sufficiently balance the low δ^15^N values of termites. Nitrogenous compounds (termite saliva) incorporated into mounds walls or gases emitted to the atmosphere could provide additional sinks for heavy nitrogen isotopes. The analyzed wall structures of queen chambers were found to be slightly enriched in ^15^N compared to fungus combs and nodules, but their nitrogen content was very low (Fig. 2). Total amount of nitrogen incorporated in nest soil structures needs to be studied in more detailed to properly evaluate their role in the colony nitrogen balance. Also the isotopic compositions of nitrogenous gas emissions, e.g. N_2_O, produced by colonies of Macrotermitinae^75^ remains to be quantified in future studies.

An alternative, or complementary, hypothesis for explaining both the very low δ^15^N values of the queens and the overall low δ^15^N levels of termite biomass relative to plant sources would be the fixation of atmospheric nitrogen (δ^15^N = ca. 0‰) by symbiotic bacteria within termite guts. Nitrogen fixation is common among termites, and gut symbionts capable to N_2_ fixation occur in many termite groups including also fungus-growing termites^76,77^. It has been suggested that the proportion of atmospheric nitrogen might exceed 50% in tissues of some wood-feeding termite species^78^. Although it has been generally thought that microbial nitrogen fixation would not play an essential role in ecology of fungus-growing termites, as the C/N balance of their food is already improved by the symbiotic *Termitomyces*^4,77^, occurrence of active nitrogen fixing bacteria would conveniently account for the unrecognized flux of isotopically light nitrogen that is required to correct the imbalance in nitrogen isotope ratios revealed by our results.

The high δ^15^N values of worker gut samples compared to plant matter in food storages and fungus combs does not support the idea that significant amounts of nitrogen fixation would take place in the guts of termite workers. Instead, we suggest that nitrogen fixation might occur within the body of the termite queen. Although no information is presently available on microbiota of termite queens, the presence of active nitrogen fixing bacteria within queen bodies seems plausible or perhaps even likely.

Firstly, queens and eggs display the largest isotopic imbalance, i.e. depletion in ^15^N compared to assumed principal food source. Although the low δ^15^N values might be partially explained by UA recycling as discussed earlier, a constant supply of isotopically light atmospheric nitrogen would provide a much more satisfying explanation for the observed ^15^N depletion pattern. Secondly, the nitrogen demand of a physogastric *Macrotermes* queen is enormous. For example, a large *M*. *michaelseni* queen weighing 20 g produces approximately 11 500 eggs per day^37^ corresponding to a biomass of ca. 350 mg^79^ which, in turn, contains ca. 28 mg of nitrogen (N content of eggs 8.5%). To accumulate the required amount of nitrogen, the queen would need to consume as many as 1750 *Termitomyces* nodules daily (N content: 8%, dry mass: 0.2 mg). Considering that fungal nodules represented the most proteinaceous food source abundantly available in the nest environment, it seems unlikely that nitrogen from dietary sources alone could sufficiently compensate the constant nitrogen loss of the queens. The uric acid stored within the fat bodies of old termite workers would represent a more nitrogenous food source than fungal nodules, but based on field observations, the availability of such workers seems to be limited. Thus, the potential occurrence of nitrogen fixation in *Macrotermes* queens should be addressed in future studies.

In sharp contrast to the queens, the δ^15^N values of termite kings were consistently higher than those recorded from any other caste. The explanation for this might be largely linked to the different origins of nitrogen incorporated in their tissues. While δ^15^N values of all other termites within the colony may be strongly impacted by mechanisms that are in place to supply the queen with enough nitrogen (i.e. accumulation and recycling of UA and/or potential N_2_ fixation), the diet of the king probably only includes nitrogen originating from plant or fungal material. The higher δ^15^N values of the kings compared to adult sterile castes (with corresponding diet) could reflect their dramatic difference in age: in mature colonies, all tissues of the king have been regenerated innumerable times, whereas the chitin and proteins of all other, much younger sterile castes, still mostly consist of relatively ^15^N depleted nitrogen incorporated during larval stages of development. In contrast, repeated regeneration of chitin and proteins probably act to continuously increase the δ^15^N values of kings from the level of plant material, fungus combs and nodules, as transamination during tissue recycling and regeneration is known to generally discriminate light nitrogen isotopes^67^.

## Material and Methods

### Sampling of termite colonies

Four termite mounds were excavated in Taita Taveta, Southern Kenya. Two of the colonies (TR9, TR183) were identified as *Macrotermes subhyalinus* and the remaining two (TR400, MR1) as *M*. *michaelseni* based on mound type (i.e. open vs. closed ventilation^80–83^). Colonies TR9, TR183 and TR400 were located at woodland savanna with large *Commiphora* and *Acacia* trees (Kasigau Road), whereas the colony MR1 was situated in relatively dense bushland (Mbula)^83^. Colonies at Kasigau Road were sampled in October 2018 whereas the colony MR1 in Mbula was sampled in January 2018.

Fungus comb material, several fungal nodules and termites representing all available castes were collected from two distinct fungus chambers from opposite sides of each nest. Termite specimens and nodules were first preserved in absolute ethanol, whereas combs were stored in paper bags. The queen chamber of each nest was removed and carefully opened (Supplementary Fig. S2), after which the queen, king and several nursing workers were collected in absolute ethanol. Larvae were collected from galleries near the queen chamber (Supplementary Fig. S3). In addition, wall material was sampled from each queen chamber. Building workers were sampled separately from outside the chambers (Supplementary Fig. S3). Nymphs (supposed to represent 5th instar occurring in Kenyan *M*. *michaelseni* colonies in October^84^) were found and collected from colony TR400. Few minor workers with notably whitish and swollen abdomens were found from the bottom parts of colony TR400 (Supplementary Fig. S3). These minor workers, probably representing old individuals with high amounts of accumulated uric acid in their abdomens^46^, were collected separately from other minor workers. Food storage material (consisting of 1–6 mm pieces of leaf and wood tissues) was identified and collected from colonies TR9 and TR400. Respectively, fecal material was found and sampled from colonies TR9 and TR183 but was not detected from the other nests.

In addition to material obtained from the termite nests, plant specimens (including most common grasses and several different tree/shrub species) were collected mostly from the immediate vicinity of the studied mounds. However, as only negligible amounts of grasses were available at the Kasigau Road study area during sampling, most grass specimens were sampled from neighboring grassland areas in Taita Hills Wildlife Sanctuary (Supplementary Table S1).

Later during the day of collection, all termite specimens originating from different nest locations were further sorted into different castes, including minor and major workers, minor and major soldiers and larvae. Larvae were divided into two groups based on their size: ‘early instars’ and ‘later instars’ (Supplementary Fig. S3). In addition, a few minor presoldiers were identified from colony TR400 (Supplementary Fig. S3). The sorted groups of different castes and all the other specimens were dried overnight at +40 °C.

### Sample preparation

Several major and minor workers (from colonies TR9, TR183 and TR400) representing different nest locations and tasks (fungal chambers, queen chambers, builders) were dissected under a stereo microscope. Gut content was separated from the abdomens and inspected by cutting the hindgut with sharp knife and tweezers. Guts including yellowish material with relatively long and clearly identifiable plant fibers were interpreted as primary food (i.e. the content of first gut passage) (Supplementary Fig. S5). Correspondingly, all guts including dark brown and relatively solid material with clearly visible soil particles were identified as secondary food (i.e. the content of second gut passage) (Supplementary Fig. S5). Two different types of guts were always pooled into two separate samples, each representing 2–10 individuals. Heads of the dissected workers were respectively collected and pooled into distinct samples. Alimentary tracts of some dissected individuals were surrounded by notable amounts of white chalk-like powder, presumably fat bodies rich in uric acid. This was especially evident for the distinctive group of minor workers collected from the colony TR400 having whitish and swollen abdomens. These fat body tissues were collected and pooled into one sample from a few individuals per colony.

Soldiers, larvae, nymphs, and those workers that were not subjected under microscopic exploration were analyzed as a whole, each sample always consisting of several individuals. The queens of the colonies TR9, TR183 and TR400 were divided into sub-samples to analyze separately their different body parts. Queen bodies were first longitudinally divided into two pieces. One side was used as a sample of entire body, whereas the other side was further divided into two parts (abdomen and head/thorax), thus leading to a total of three different sub-samples from each queen. Queen of the colony MR1 was analyzed only as a whole. Small proportion of each queen sample including abdominal tissues was subjected to lipid removal treatment (see Supplementary Methods).

Topmost rims, often differing in color from the other parts (Supplementary Fig. S4), were scratched off from the fungus combs and represented samples of fresh comb material, whereas several pieces collected from the lower comb parts were pooled to represent old comb material. Several (>10) fungal nodules were always pooled to represent one sample (Supplementary Fig. S4). Food storage material was rinsed and floated several times in MQ to separate plant pieces from mineral soil. Final fecal material was rinsed with MQ and separated from soil under a stereo microscope.

Dry samples were homogenized either manually using an agate mortar and pestle (termites, fungus combs, nodules, fecal material), or cryo-milled with liquid N_2_ cooling (plant matter), and weighed in tin cups.

### Analysis of stable isotopes

The isotopic composition and content of carbon and nitrogen was measured on a NC2500 elemental analyzer coupled to a Thermo Scientific Delta V Plus isotope ratio mass spectrometer at the Laboratory of Chronology, Finnish Museum of Natural History. Low C/N ratio samples (termites, fungal nodules) were analyzed in a dual analysis mode, where both C and N data are derived from a single sample. Higher C/N ratio samples (plants, fungus combs, and final feces) were analyzed in two analytical runs, separately for carbon and nitrogen, respectively. The raw isotope data was normalized with a multi-point calibration using certified isotopic reference materials (USGS-40, USGS-41, IAEA-N1, IAEA-N2, IAEA-CH3 and IAEA-CH7). Duplicate or quadruplicate analyses of subsamples placed consecutively within the analytical sequence yielded a reproducibility of ≤0.1‰ for both δ^13^C and δ^15^N values. Measurements of quality control reference materials over the entire analytical period indicate an internal precision of ≤0.2‰ for both δ^13^C and δ^15^N.

## Supplementary information

Supplementary results and methods

## Data Availability

All datasets produced during this study are provided in the article and in Supplementary Tables S1 and S2.
