# Supplementary material for: Caste-specific nutritional differences define carbon and nitrogen fluxes within symbiotic food webs in African termite mounds
Source: Sci Rep. 2019 Nov 13;9:16698. doi: 10.1038/s41598-019-53153-x (PMC6853943; doi:10.1038/s41598-019-53153-x)
Supplement: Supplementary file 1 — Supplementary results and methods [file 41598_2019_53153_MOESM1_ESM.docx]

**Caste-specific nutritional differences define carbon and nitrogen fluxes within symbiotic food webs in African termite mounds**

Risto Vesala, Laura Arppe, Jouko Rikkinen

# Supplementary results

**Supplementary Table S1.** Stable isotope values (δ^13^C and δ^15^N), carbon and nitrogen contents and C/N ratios of plant specimens collected either from the Kasigau Road study area or Taita Hills Wildlife Sanctuary (THWS).

| **Sampling location** | **Plant type** | **Species** | **Plant part** | **δ^13^C** | **δ^15^N** | **C cont. (%)** | **N cont. (%)** | **C/N ratio** |
| --- | --- | --- | --- | --- | --- | --- | --- | --- |
| Kasigau Road | tree/shrub | *Acacia mellifera* | Bark | -28.0 | -1.6 | 46.0 | 0.7 | 70.8 |
| Kasigau Road | tree/shrub | *Acacia mellifera* | Leaves (green) | -29.3 | 6.6 | 42.2 | 2.7 | 15.5 |
| Kasigau Road | tree/shrub | *Acacia mellifera* | Wood | -26.6 | -1.4 | 45.0 | 0.4 | 112.5 |
| Kasigau Road | tree/shrub | *Acacia tortilis* | Bark | -26.2 | -1.4 | 44.7 | 1.6 | 28.1 |
| THWS | tree/shrub | *Acacia tortilis* | Leaves (green) | -29.2 | 3.4 | 47.4 | 3.7 | 13.0 |
| THWS | tree/shrub | *Acacia tortilis* | Leaves (green) | -26.7 | 0.4 | 50.0 | 4.8 | 10.4 |
| Kasigau Road | tree/shrub | *Acacia tortilis* | Wood | -28.3 | 3.2 | 46.5 | 1.0 | 45.2 |
| Kasigau Road | tree/shrub | *Acacia tortilis* | Wood | -26.3 | -0.6 | 48.7 | 0.6 | 81.2 |
| THWS | tree/shrub | *Acacia tortilis* | Wood | -28.7 | 1.9 | 44.3 | 1.3 | 33.8 |
| THWS | tree/shrub | *Acacia tortilis* | Wood | -28.0 | -0.2 | 46.4 | 1.4 | 32.2 |
| THWS | tree/shrub | *Balanites aegyptiaca* | Bark | -25.6 | 9.2 | 42.1 | 1.8 | 23.3 |
| THWS | tree/shrub | *Balanites aegyptiaca* | Leaves (green) | -27.4 | 9.5 | 43.7 | 2.2 | 19.7 |
| THWS | tree/shrub | *Balanites aegyptiaca* | Leaves (green) | -27.1 | 6.2 | 42.1 | 2.8 | 15.0 |
| THWS | tree/shrub | *Balanites aegyptiaca* | Wood | -26.5 | 3.6 | 48.6 | 1.6 | 30.4 |
| THWS | tree/shrub | *Bourreria teitensis* | Leaves (green) | -27.6 | 7.9 | 31.9 | 1.3 | 25.3 |
| THWS | tree/shrub | *Bourreria teitensis* | Wood | -27.2 | 6.4 | 45.7 | 1.1 | 40.1 |
| Kasigau Road | grass | *Chlorix roxburghiana* | Leaves (brown) | -14.7 | 4.1 | 43.8 | 1.3 | 32.9 |
| Kasigau Road | grass | *Chlorix roxburghiana* | Leaves (brown) | -13.4 | 2.0 | 40.6 | 1.1 | 36.6 |
| Kasigau Road | grass | *Chlorix roxburghiana* | Leaves (brown) | -14.5 | 5.3 | 43.3 | 1.2 | 36.1 |
| THWS | grass | *Chlorix roxburghiana* | Leaves (brown) | -13.7 | 7.7 | 38.8 | 1.7 | 23.5 |
| THWS | grass | *Chlorix roxburghiana* | Leaves (brown) | -11.9 | 3.8 | 40.9 | 1.5 | 27.8 |
| THWS | grass | *Chlorix roxburghiana* | Leaves (brown) | -12.9 | 6.6 | 39.8 | 1.7 | 23.8 |
| THWS | grass | *Chlorix roxburghiana* | Leaves (brown) | -12.9 | 5.4 | 39.8 | 0.8 | 50.4 |
| THWS | grass | *Chlorix roxburghiana* | Leaves (brown) | -13.4 | 3.3 | 40.1 | 0.9 | 46.6 |
| Kasigau Road | grass | *Chlorix roxburghiana* | Leaves (green) | -14.5 | 4.8 | 42.4 | 1.5 | 29.2 |
| THWS | grass | *Chlorix roxburghiana* | Leaves (green) | -13.6 | 6.2 | 39.9 | 1.1 | 37.6 |
| THWS | grass | *Chlorix roxburghiana* | Leaves (green) | -14.7 | 3.7 | 41.7 | 0.8 | 55.6 |
| Kasigau Road | tree/shrub | *Commiphora sp.* | Bark | -26.1 | 3.7 | 48.7 | 0.7 | 67.6 |
| Kasigau Road | tree/shrub | *Commiphora sp.* | Bark | -29.3 | 0.2 | 47.4 | 0.5 | 100.9 |
| Kasigau Road | tree/shrub | *Commiphora sp.* | Leaves (brown) | -29.1 | 6.7 | 44.4 | 2.0 | 21.8 |
| Kasigau Road | tree/shrub | *Commiphora sp.* | Leaves (green) | -25.8 | 5.9 | 47.5 | 2.3 | 20.7 |
| Kasigau Road | tree/shrub | *Commiphora sp.* | Wood | -28.0 | 5.2 | 45.2 | 0.8 | 55.8 |
| Maktau | grass | *Cynodon dactylon* | Leaves (brown) | -14.5 | 7.2 | 40.2 | 3.3 | 12.2 |
| THWS | grass | *Cynodon dactylon* | Leaves (brown) | -13.2 | 7.2 | 39.3 | 1.3 | 31.4 |
| THWS | grass | *Cynodon dactylon* | Leaves (brown) | -12.8 | 3.4 | 41.5 | 0.6 | 74.1 |
| THWS | grass | *Cynodon dactylon* | Leaves (brown) | -13.2 | 5.1 | 40.7 | 1.2 | 35.1 |
| THWS | grass | *Cynodon dactylon* | Leaves (green) | -14.2 | 9.1 | 40.4 | 3.1 | 13.1 |
| THWS | grass | *Cynodon dactylon* | Leaves (green) | -13.7 | 6.9 | 39.3 | 2.1 | 18.5 |
| Maktau | grass | *Enteropogon macrostachyus* | Leaves (brown) | -14.5 | 5.8 | 42.0 | 1.8 | 23.2 |
| THWS | tree/shrub | *Grewia villosa* | Leaves (green) | -29.3 | 5.5 | 44.5 | 2.5 | 17.7 |
| THWS | tree/shrub | *Grewia villosa* | Leaves (green) | -28.1 | 5.3 | 42.1 | 2.7 | 15.7 |
| THWS | tree/shrub | *Grewia villosa* | Wood | -27.3 | 2.3 | 43.5 | 1.0 | 43.9 |
| THWS | tree/shrub | *Grewia villosa* | Wood | -27.1 | 4.8 | 43.9 | 0.9 | 49.9 |
| THWS | grass | Not identified | Leaves (brown) | -13.8 | 3.6 | 41.7 | 0.8 | 53.5 |
| THWS | grass | Not identified | Leaves (brown) | -12.2 | 6.3 | 38.4 | 0.9 | 42.7 |
| THWS | tree/shrub | *Salvadora persica* | Bark | -26.0 | 4.7 | 25.7 | 2.5 | 10.1 |
| THWS | tree/shrub | *Salvadora persica* | Leaves (green) | -25.3 | 8.0 | 34.3 | 3.5 | 9.9 |
| THWS | tree/shrub | *Salvadora persica* | Wood | -22.3 | 3.6 | 44.7 | 0.9 | 52.6 |
| THWS | grass | *Themeda triandra* | Leaves (brown) | -12.7 | 2.4 | 40.8 | 0.6 | 69.2 |

**Supplementary Table S2.** Stable isotope values (δ^13^C and δ^15^N), carbon and nitrogen contents and C/N ratios of all analyzed materials collected from termite nests. Chambers: A, B, C = different fungus chambers, Q = queen chamber, empty = other nest locations (e.g. workers repairing nest walls).

| **Colony** | **Chamber** | **Sample description** | **δ13C** | **δ15N** | **C cont. (%)** | **N cont. (%)** | **C/N ratio** |
| --- | --- | --- | --- | --- | --- | --- | --- |
| MR1 | A | Fresh fungus comb | -24.1 | 6.8 | 41.3 | 2.3 | 18.2 |
| MR1 | C | Fresh fungus comb | -24.5 | 6.6 | 44.8 | 1.9 | 23.7 |
| MR1 | A | Old fungus comb | -21.4 | 6.5 | 40.8 | 1.8 | 23.4 |
| MR1 | C | Old fungus comb | -24.0 | 6.7 | 44.3 | 1.8 | 25.1 |
| MR1 | A | Fungal nodules | -20.3 | 6.9 | 44.7 | 7.0 | 6.4 |
| MR1 | C | Fungal nodules | -20.7 | 6.7 | 44.7 | 7.8 | 5.7 |
| MR1 | A | Larvae, early instars | -21.3 | 5.3 | 46.4 | 11.0 | 4.2 |
| MR1 | C | Larvae, early instars | -21.5 | 5.4 | 47.4 | 10.8 | 4.4 |
| MR1 | A | Larvae, late instars | -21.8 | 5.6 | 48.1 | 10.9 | 4.4 |
| MR1 | C | Larvae, late instars | -21.2 | 6.0 | 47.3 | 11.3 | 4.2 |
| MR1 | A | Workers (major and minor) | -21.9 | 5.7 | 29.4 | 5.8 | 5.1 |
| MR1 | C | Workers (major and minor) | -21.9 | 5.7 | 31.6 | 6.1 | 5.2 |
| MR1 | A | Major soldiers | -21.2 | 5.8 | 49.8 | 10.8 | 4.6 |
| MR1 | C | Major soldiers | -21.4 | 6.0 | 52.5 | 11.1 | 4.7 |
| MR1 | A | Minor soldiers | -20.8 | 6.0 | 47.4 | 11.5 | 4.1 |
| MR1 | C | Minor soldiers | -20.9 | 5.9 | 47.4 | 11.2 | 4.2 |
| MR1 | Q | King | -21.8 | 8.0 | 57.6 | 8.7 | 6.6 |
| MR1 | Q | Queen, whole body | -21.9 | 4.8 | 56.4 | 8.3 | 6.8 |
| MR1 | Q | Queen, whole body (lipid treatment) | -19.7 | 5.3 | 48.7 | 13.8 | 3.5 |
| TR183 | A | Fresh fungus comb | -25.4 | 4.6 | 44.2 | 1.9 | 23.1 |
| TR183 | B | Fresh fungus comb | -27.1 | 4.9 | 41.5 | 3.0 | 13.9 |
| TR183 | A | Old fungus comb | -26.4 | 4.9 | 40.3 | 2.6 | 15.7 |
| TR183 | B | Old fungus comb | -26.4 | 4.7 | 41.3 | 2.8 | 14.6 |
| TR183 | A | Fungal nodules | -23.6 | 4.6 | 41.4 | 6.9 | 6.0 |
| TR183 | B | Fungal nodules | -23.4 | 4.6 | 43.5 | 7.3 | 6.0 |
| TR183 | Q | Larvae, early instars | -24.0 | 3.6 | 44.3 | 10.9 | 4.1 |
| TR183 | A | Larvae, late instars | -24.7 | 3.8 | 45.8 | 9.9 | 4.6 |
| TR183 | B | Larvae, late instars | -24.8 | 3.7 | 46.1 | 9.9 | 4.7 |
| TR183 | Q | Larvae, late instars | -24.6 | 3.5 | 45.4 | 9.9 | 4.6 |
| TR183 | A | Major workers | -25.2 | 4.3 | 44.2 | 8.6 | 5.1 |
| TR183 | B | Major workers | -25.5 | 4.3 | 44.1 | 9.0 | 4.9 |
| TR183 | Q | Major workers | -25.5 | 4.0 | 39.9 | 8.5 | 4.7 |
| TR183 | A | Minor workers | -25.3 | 3.9 | 45.5 | 10.8 | 4.2 |
| TR183 | B | Minor workers | -25.9 | 4.2 | 40.9 | 7.0 | 5.8 |
| TR183 | Q | Minor workers | -26.7 | 4.5 | 42.5 | 6.7 | 6.3 |
| TR183 | A | Major soldiers | -26.0 | 3.9 | 49.1 | 10.7 | 4.6 |
| TR183 | B | Major soldiers | -26.0 | 3.8 | 50.6 | 11.3 | 4.5 |
| TR183 | A | Minor soldiers | -25.4 | 4.5 | 45.4 | 11.2 | 4.1 |
| TR183 | B | Minor soldiers | -25.4 | 4.3 | 46.6 | 11.4 | 4.1 |
| TR183 | Q | King | -25.1 | 5.6 | 56.7 | 7.8 | 7.3 |
| TR183 | Q | Queen, abdomen | -25.7 | 3.4 | 53.4 | 8.5 | 6.3 |
| TR183 | Q | Queen, abdomen (lipid treatment) | -23.3 | 3.6 | 49.7 | 14.4 | 3.4 |
| TR183 | Q | Queen, head | -23.0 | 3.5 | 51.2 | 10.3 | 5.0 |
| TR183 | Q | Queen, whole body | -25.6 | 3.3 | 53.4 | 8.1 | 6.6 |
| TR183 | Q | Queen, whole body (lipid treatment) | -23.3 | 3.6 | 49.8 | 14.6 | 3.4 |
| TR183 | Q | Guts of major workes (passage 1) | -27.0 | 6.1 | 32.4 | 2.7 | 12.0 |
| TR183 |  | Guts of major workes (passage 1) | -26.5 | 6.1 | 31.3 | 3.2 | 9.9 |
| TR183 |  | Guts of major workes (passage 2) | -26.7 | 4.6 | 30.6 | 4.0 | 7.6 |
| TR183 | B | Guts of minor workes (passage 1) | -27.4 | 5.1 | 36.9 | 4.5 | 8.2 |
| TR183 | Q | Guts of minor workes (passage 1) | -27.6 | 6.1 | 38.4 | 3.6 | 10.7 |
| TR183 | A | Guts of minor workes (passage 2) | -27.0 | 4.3 | 39.9 | 5.6 | 7.1 |
| TR183 | Q | Heads of major workers | -25.2 | 4.4 | 48.0 | 11.5 | 4.2 |
| TR183 |  | Heads of major workers | -25.2 | 4.4 | 47.9 | 11.9 | 4.0 |
| TR183 | A | Heads of minor workers | -25.5 | 3.9 | 47.0 | 11.4 | 4.1 |
| TR183 | B | Heads of minor workers | -25.4 | 4.4 | 47.6 | 11.3 | 4.2 |
| TR183 | Q | Heads of minor workers | -25.4 | 4.4 | 47.2 | 11.2 | 4.2 |
| TR183 | Q | Fat bodies of workers (rich in UA) | -25.3 | 2.8 | 39.3 | 5.4 | 7.3 |
| TR183 |  | Final feces | -25.1 | 6.0 | 7.2 | 0.5 | 13.8 |
| TR183 |  | Nest (queen chamber) wall | -24.1 | 4.9 | 1.5 | 0.1 | 11.8 |
| TR400 | A | Fresh fungus comb | -25.6 | 4.9 | 42.9 | 2.9 | 14.6 |
| TR400 | B | Fresh fungus comb | -25.1 | 5.1 | 41.1 | 2.5 | 16.7 |
| TR400 | A | Old fungus comb | -25.6 | 4.3 | 42.3 | 2.1 | 20.5 |
| TR400 | B | Old fungus comb | -25.5 | 5.0 | 41.5 | 2.4 | 17.5 |
| TR400 | A | Fungal nodules | -22.5 | 5.2 | 45.5 | 8.8 | 5.2 |
| TR400 | B | Fungal nodules | -23.1 | 5.4 | 45.2 | 8.5 | 5.4 |
| TR400 | Q | Eggs | -24.2 | 4.5 | 50.6 | 8.5 | 6.0 |
| TR400 |  | Nymphs | -26.8 | 4.0 | 68.4 | 5.4 | 12.6 |
| TR400 | Q | Larvae, early instars | -23.7 | 4.2 | 45.1 | 10.1 | 4.5 |
| TR400 | A | Larvae, late instars | -23.8 | 4.7 | 45.1 | 11.1 | 4.1 |
| TR400 | B | Larvae, late instars | -24.0 | 4.8 | 46.1 | 10.5 | 4.4 |
| TR400 | A | Major workers | -25.2 | 4.1 | 37.1 | 8.5 | 4.4 |
| TR400 | B | Major workers | -25.3 | 4.1 | 36.4 | 8.1 | 4.5 |
| TR400 | Q | Major workers | -25.1 | 5.0 | 39.6 | 7.5 | 5.3 |
| TR400 |  | Major workers | -25.1 | 4.2 | 37.6 | 8.5 | 4.4 |
| TR400 |  | Minor workers (whitish abdomen) | -24.5 | 0.8 | 35.7 | 18.5 | 1.9 |
| TR400 | A | Major soldiers | -26.1 | 4.7 | 50.4 | 10.3 | 4.9 |
| TR400 | B | Major soldiers | -26.1 | 3.9 | 50.7 | 11.1 | 4.6 |
| TR400 | A | Minor soldiers | -25.0 | 5.1 | 46.0 | 10.5 | 4.4 |
| TR400 | B | Minor soldiers | -25.0 | 5.0 | 46.6 | 10.8 | 4.3 |
| TR400 | B | Presoldiers | -24.2 | 4.6 | 46.9 | 11.6 | 4.1 |
| TR400 | Q | King | -25.2 | 5.9 | 56.9 | 8.1 | 7.1 |
| TR400 | Q | Queen, abdomen | -24.6 | 4.1 | 53.4 | 8.4 | 6.4 |
| TR400 | Q | Queen, abdomen (lipid treatment) | -22.8 | 4.6 | 48.6 | 14.2 | 3.4 |
| TR400 | Q | Queen, head | -23.1 | 3.5 | 49.1 | 10.3 | 4.8 |
| TR400 | Q | Queen, whole body | -24.6 | 4.0 | 54.7 | 8.5 | 6.4 |
| TR400 | Q | Queen, whole body (lipid treatment) | -22.8 | 4.1 | 48.7 | 14.4 | 3.4 |
| TR400 |  | Guts of major workers (passage 1) | -26.2 | 7.3 | 24.8 | 2.9 | 8.6 |
| TR400 | B | Guts of major workers (passage 2) | -26.3 | 5.5 | 28.9 | 3.7 | 7.9 |
| TR400 | Q | Guts of major workers (passage 2) | -25.9 | 5.2 | 33.7 | 4.5 | 7.6 |
| TR400 | B | Guts of minor workers (passage 1) | -25.5 | 5.1 | 40.1 | 5.0 | 8.1 |
| TR400 |  | Guts of minor workers (passage 1) | -26.1 | 6.3 | 24.7 | 5.3 | 4.7 |
| TR400 | B | Guts of minor workers (passage 2) | -26.2 | 3.6 | 30.1 | 4.8 | 6.2 |
| TR400 | B | Heads of major workers | -24.9 | 4.9 | 47.2 | 12.2 | 3.9 |
| TR400 | Q | Heads of major workers | -24.8 | 5.1 | 48.0 | 12.0 | 4.0 |
| TR400 |  | Heads of major workers | -24.9 | 4.9 | 46.4 | 11.9 | 3.9 |
| TR400 | B | Heads of minor workers | -24.7 | 5.1 | 46.6 | 11.8 | 3.9 |
| TR400 |  | Fat bodies of workers (rich in UA) | -23.4 | -1.0 | 32.5 | 27.3 | 1.2 |
| TR400 |  | Food storage | -28.2 | 4.4 | 47.3 | 1.8 | 26.7 |
| TR400 |  | Nest (queen chamber) wall | -20.2 | 5.1 | 1.0 | 0.1 | 8.3 |
| TR9 | A | Fresh fungus comb | -24.6 | 5.0 | 42.9 | 2.7 | 15.9 |
| TR9 | B | Fresh fungus comb | -24.3 | 5.0 | 42.7 | 2.7 | 15.9 |
| TR9 | A | Old fungus comb | -26.0 | 4.8 | 42.5 | 2.7 | 15.7 |
| TR9 | B | Old fungus comb | -26.2 | 5.1 | 40.4 | 2.9 | 14.0 |
| TR9 | A | Fungal nodules | -23.5 | 5.1 | 44.5 | 9.1 | 4.9 |
| TR9 | B | Fungal nodules | -23.1 | 5.0 | 43.6 | 7.9 | 5.6 |
| TR9 | Q | Larvae, early instars | -23.2 | 4.0 | 44.9 | 10.5 | 4.3 |
| TR9 | A | Larvae, late instars | -24.2 | 4.4 | 46.7 | 9.8 | 4.8 |
| TR9 | B | Larvae, late instars | -24.0 | 4.6 | 46.4 | 10.3 | 4.5 |
| TR9 | Q | Larvae, late instars | -23.7 | 4.2 | 45.9 | 10.0 | 4.6 |
| TR9 | A | Major workers | -25.1 | 4.8 | 42.2 | 8.3 | 5.1 |
| TR9 | B | Major workers | -25.0 | 4.8 | 44.4 | 9.2 | 4.8 |
| TR9 | B | Minor workers | -25.6 | 4.1 | 42.6 | 8.8 | 4.9 |
| TR9 | A | Major soldiers | -26.0 | 4.2 | 49.2 | 10.9 | 4.5 |
| TR9 | B | Major soldiers | -26.1 | 4.9 | 49.8 | 10.5 | 4.8 |
| TR9 | A | Minor soldiers | -25.4 | 5.3 | 45.6 | 10.9 | 4.2 |
| TR9 | B | Minor soldiers | -25.3 | 5.3 | 47.1 | 11.3 | 4.2 |
| TR9 | Q | King | -24.2 | 5.9 | 54.7 | 9.1 | 6.0 |
| TR9 | Q | Queen, abdomen | -25.0 | 3.9 | 54.6 | 8.0 | 6.9 |
| TR9 | Q | Queen, abdomen (lipid treatment) | -22.5 | 3.7 | 48.6 | 13.2 | 3.7 |
| TR9 | Q | Queen, head | -23.6 | 3.1 | 52.9 | 9.5 | 5.6 |
| TR9 | Q | Queen, whole body | -24.9 | 3.7 | 55.7 | 8.4 | 6.7 |
| TR9 | Q | Queen, whole body (lipid treatment) | -22.4 | 4.1 | 48.8 | 13.5 | 3.6 |
| TR9 | B | Guts of major workers (passage 1) | -25.6 | 5.8 | 40.4 | 4.6 | 8.8 |
| TR9 |  | Guts of major workers (passage 1) | -26.5 | 7.9 | 27.2 | 2.8 | 9.9 |
| TR9 | B | Guts of minor workers (passage 1) | -26.6 | 5.6 | 39.3 | 5.4 | 7.3 |
| TR9 | B | Heads of major workers | -24.9 | 4.9 | 47.9 | 11.8 | 4.1 |
| TR9 | Q | Heads of major workers | -25.1 | 4.9 | 47.1 | 12.0 | 3.9 |
| TR9 | B | Heads of minor workers | -25.4 | 4.9 | 47.6 | 11.5 | 4.1 |
| TR9 | Q | Fat bodies of workers (rich in UA) | -24.9 | 3.4 | 40.2 | 11.5 | 3.5 |
| TR9 |  | Final feces | -27.2 | 5.7 | 18.7 | 1.1 | 17.2 |
| TR9 |  | Food storage | -29.1 | 5.1 | 45.1 | 3.0 | 15.3 |
| TR9 |  | Nest (queen chamber) wall | -20.0 | 5.5 | 0.7 | 0.1 | 8.3 |


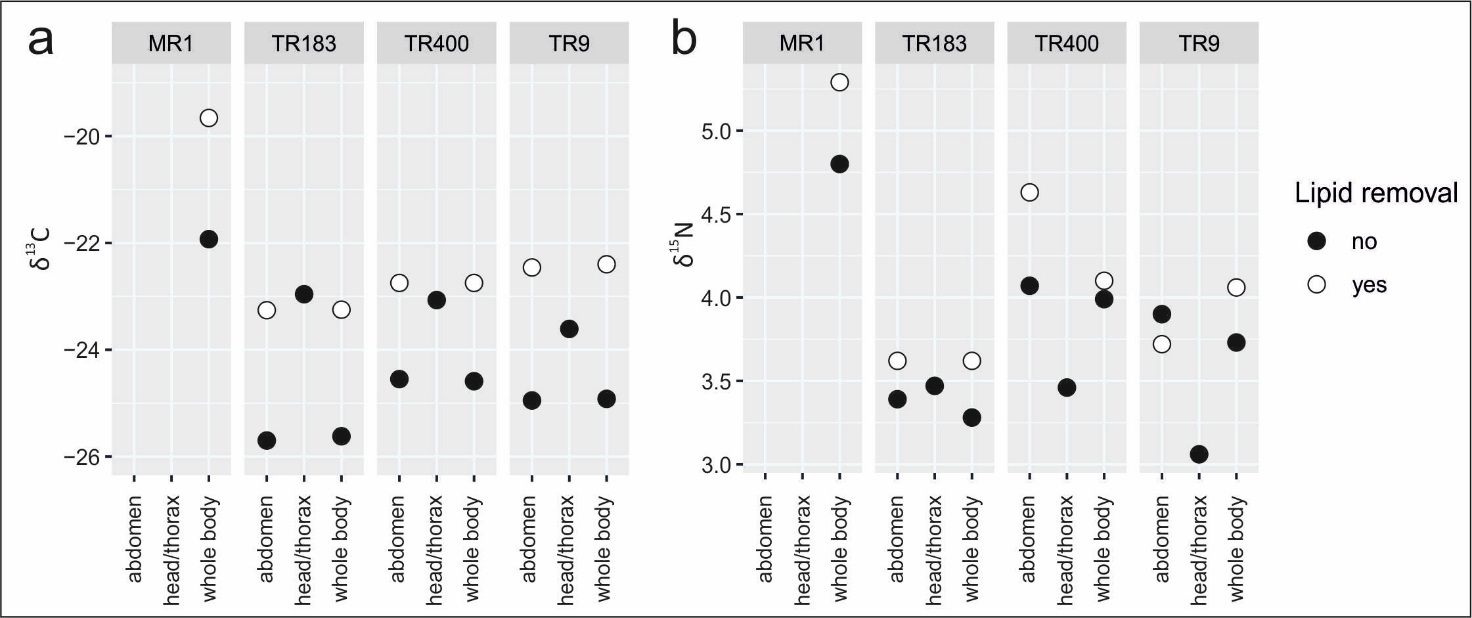


**Supplementary Figure S1.** The effect of lipid removal treatment on δ^13^C (a) and δ^15^N (b) values of the queen specimens.

# Supplementary methods

### Lipid removal of queens

For removal of lipids, subsamples of the powdered queens and king were treated with alternating washes of chloroform:methanol 2:1 and 1:2 solutions. The treatments were done in two cycles, each starting with two-three repeats with the chlo:met 2:1 and followed by chlo:met 1:2 washes. During the second treatment cycle, the samples rested in a water bath with gentle heating at 40°C. During each treatment of ~15 minutes, the vials were vortexed every five minutes and placed in a sonic bath in between to facilitate agitation. Between cycles and at the end of the process, the samples were washed with MilliQ water using the vortex and sonic agitation to enhance solvent removal five times with centrifugation in between. The number of required solution treatments, ranging from 4 to 15, were evaluated based on visual appearance of the solvent and solid sample residue. At the end of the process, samples were washed with MilliQ water as described above, and dried at 50°C.


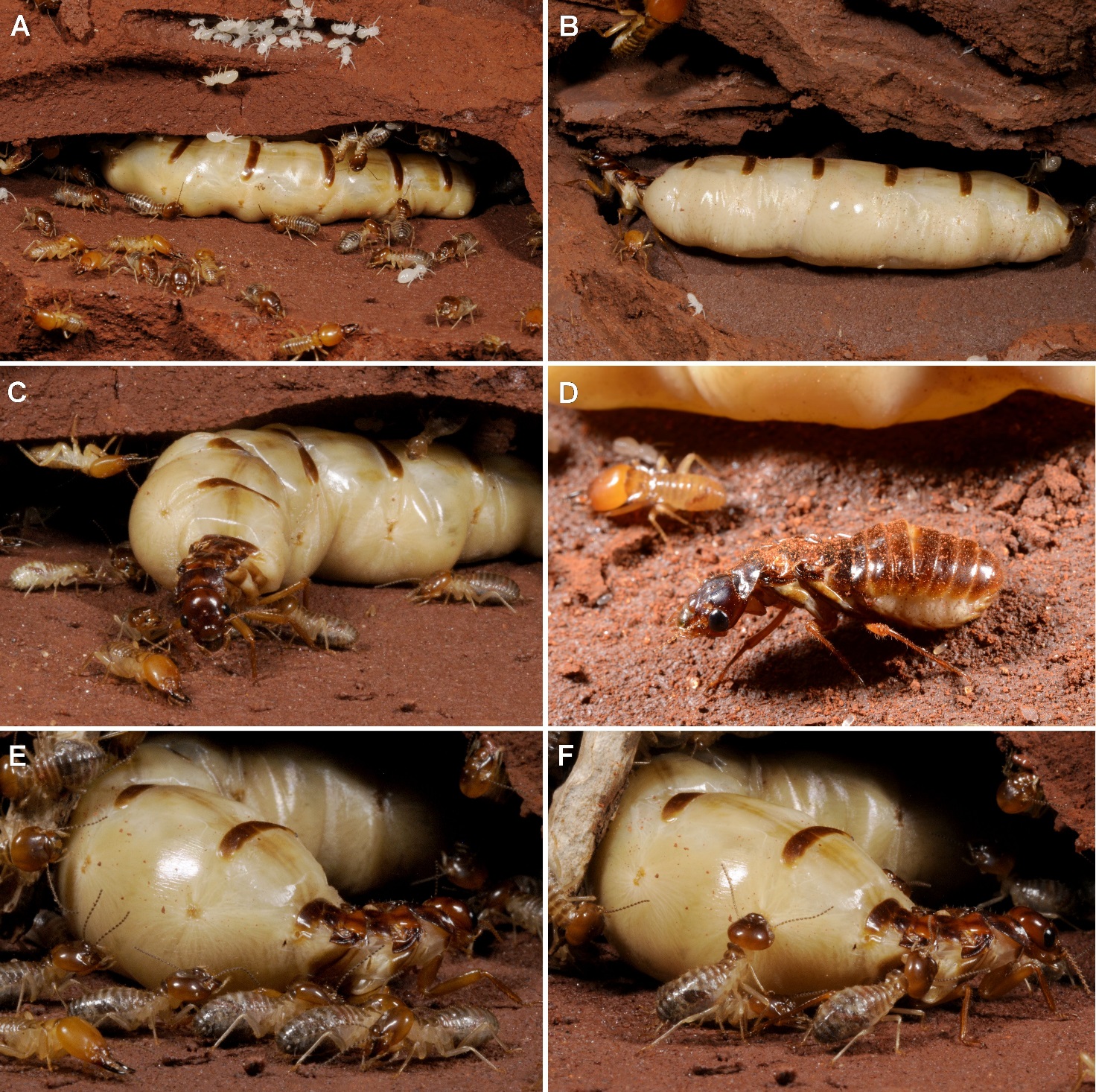


**Supplementary Figure S2.** The royal chamber of a Macrotermes colony. A. Queen and sterile castes in opened royal chamber. B. Queen. C. Front view of the queen showing the head and thorax. D. King.


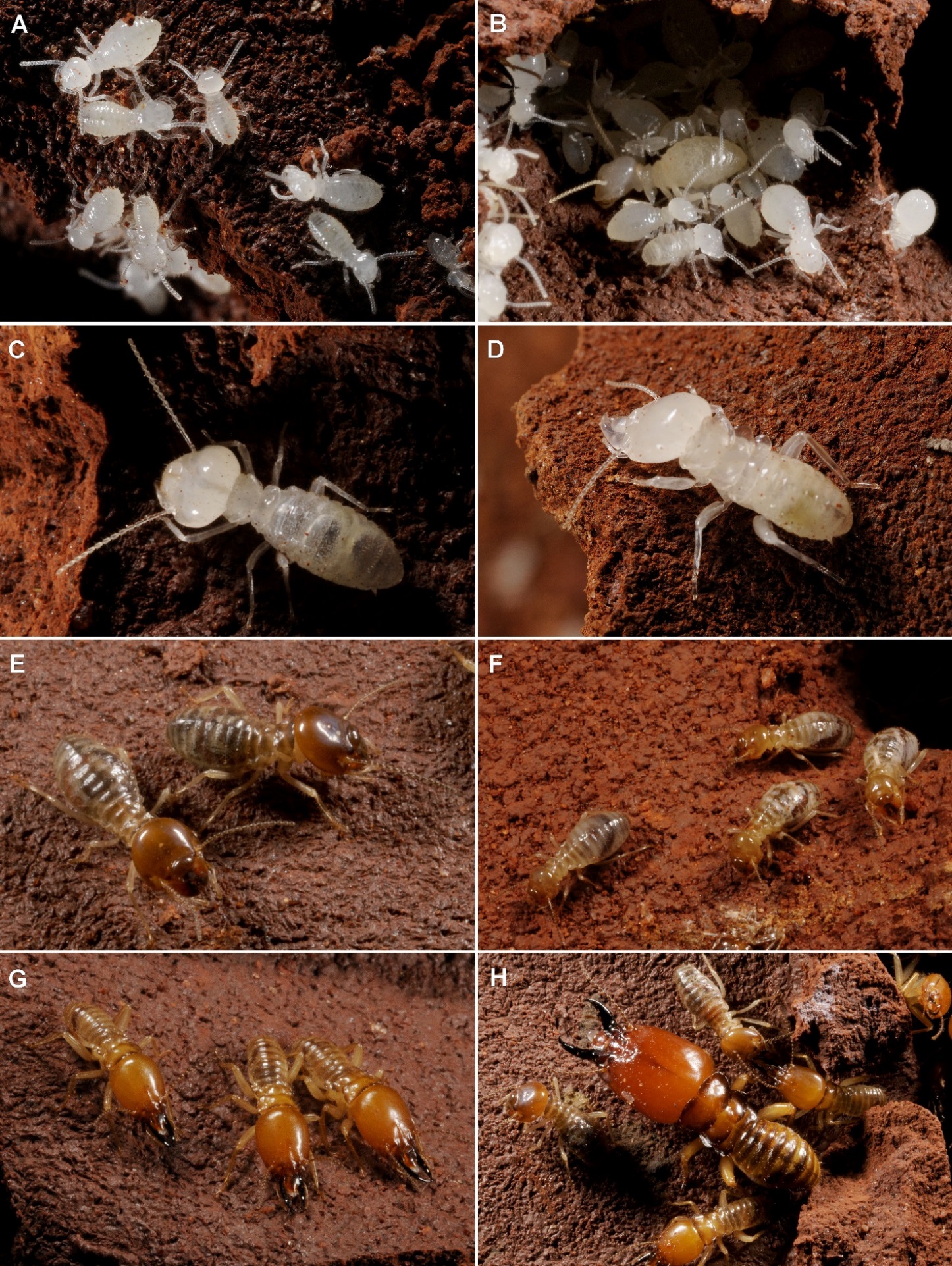


**Supplementary Figure S3.** Sterile termite castes within a *Macrotermes* colony. A. Early instar larvae. B. Early and late instar larvae. C. Late instar larvae (almost mature worker). D. Pre-soldier. E. Major workers in royal chamber. F. Minor workers. The three rightmost have accumulated visible amounts of uric acid in fat body. G. Minor soldiers. H. Major soldier with workers and minor soldiers.


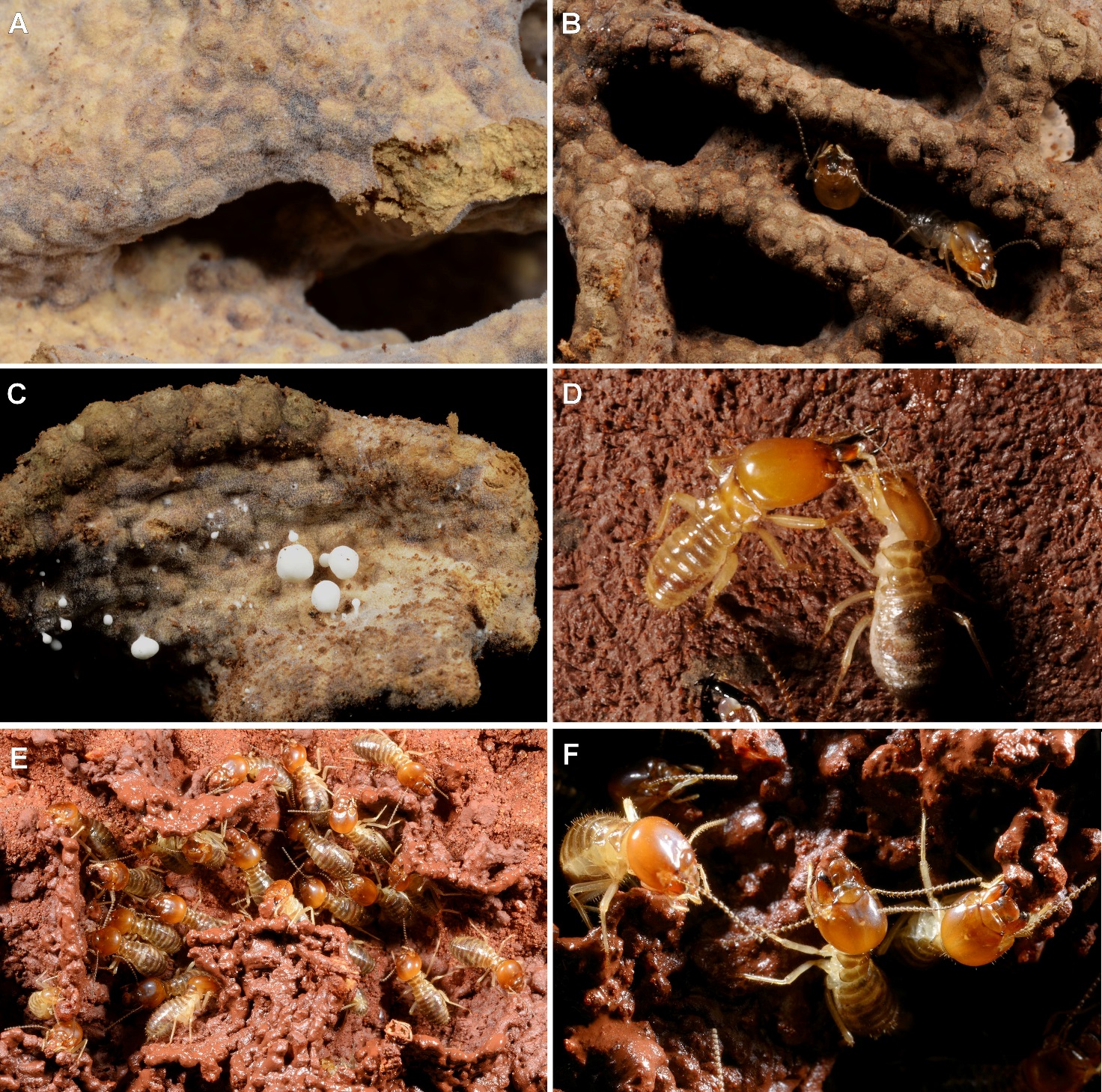


**Supplementary Figure S4.** Structures and activities within a *Macrotermes* colony. A. Fresh topmost edges of fungus comb. B. Older parts of fungus comb. C. *Termitomyces* nodules developing on comb surface. D. Worker feeding minor soldier. E and F. Workers repairing nest chamber structures with mineral soil glued together with saliva.

**
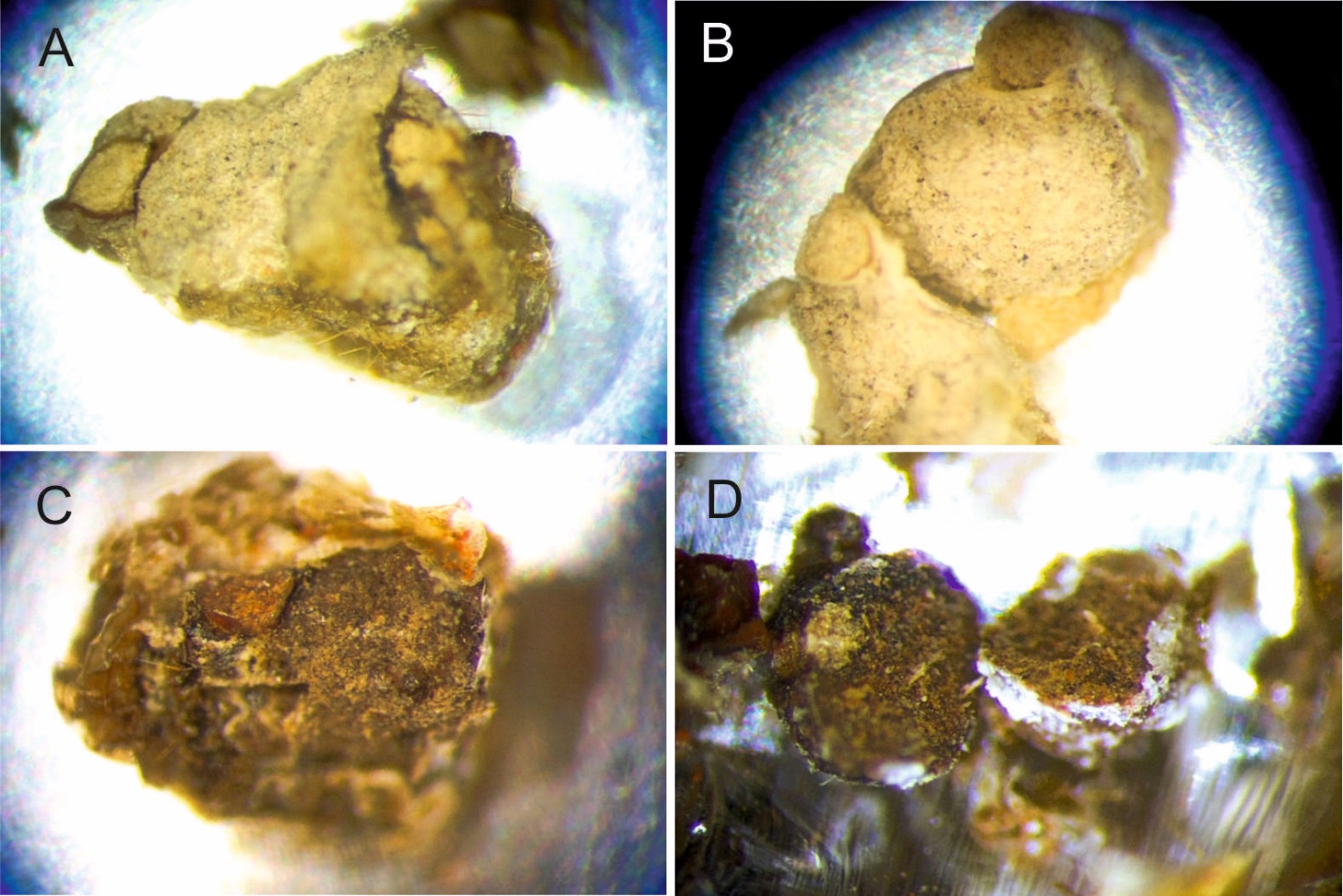
**

**Supplementary Figure S5.** Examples of gut contents of worker termites. The guts including yellowish material with relatively long and clearly identifiable plant fibers (A, B) were interpreted as primary food (i.e. the content of first gut passage), whereas the guts including dark brown and relatively solid material with clearly visible soil particles (C, D) were identified as secondary food (i.e. the content of second gut passage).
